# Incorporating genomic, transcriptomic and clinical data: a prognostic and stem cell-like MYC and PRC imbalance in high-risk neuroblastoma

**DOI:** 10.1186/s12918-017-0466-5

**Published:** 2017-10-03

**Authors:** Xinan Holly Yang, Fangming Tang, Jisu Shin, John M. Cunningham

**Affiliations:** Section of Hematology and Oncology, Departments of Pediatrics, University of Chicago, Chicago, IL 60637 USA

**Keywords:** Neuroblastoma, MYC, PRC2, ZFHX3, Somatic mutation

## Abstract

**Background:**

Previous studies suggested that cancer cells possess traits reminiscent of the biological mechanisms ascribed to normal embryonic stem cells (ESCs) regulated by MYC and Polycomb repressive complex 2 (PRC2). Several poorly differentiated adult tumors showed preferentially high expression levels in targets of MYC, coincident with low expression levels in targets of PRC2. This paper will reveal this ESC-like cancer signature in high-risk neuroblastoma (HR-NB), the most common extracranial solid tumor in children.

**Methods:**

We systematically assembled genomic variants, gene expression changes, priori knowledge of gene functions, and clinical outcomes to identify prognostic multigene signatures. First, we assigned a new, individualized prognostic index using the relative expressions between the poor- and good-outcome signature genes. We then characterized HR-NB aggressiveness beyond these prognostic multigene signatures through the imbalanced effects of MYC and PRC2 signaling. We further analyzed Retinoic acid (RA)-induced HR-NB cells to model tumor cell differentiation. Finally, we performed in vitro validation on ZFHX3, a cell differentiation marker silenced by PRC2, and compared cell morphology changes before and after blocking PRC2 in HR-NB cells.

**Results:**

A significant concurrence existed between exons with verified variants and genes showing MYCN-dependent expression in HR-NB. From these biomarker candidates, we identified two novel prognostic gene-set pairs with multi-scale oncogenic defects. Intriguingly, MYC targets over-represented an unfavorable component of the identified prognostic signatures while PRC2 targets over-represented a favorable component. The cell cycle arrest and neuronal differentiation marker ZFHX3 was identified as one of PRC2-silenced tumor suppressor candidates. Blocking PRC2 reduced tumor cell growth and increased the mRNA expression levels of ZFHX3 in an early treatment stage. This hypothesis-driven systems bioinformatics work offered novel insights into the PRC2-mediated tumor cell growth and differentiation in neuroblastoma, which may exert oncogenic effects together with MYC regulation.

**Conclusion:**

Our results propose a prognostic effect of imbalanced MYC and PRC2 moderations in pediatric HR-NB for the first time. This study demonstrates an incorporation of genomic landscapes and transcriptomic profiles into the hypothesis-driven precision prognosis and biomarker discovery. The application of this approach to neuroblastoma, as well as other cancer more broadly, could contribute to reduced relapse and mortality rates in the long term.

**Electronic supplementary material:**

The online version of this article (doi:10.1186/s12918-017-0466-5) contains supplementary material, which is available to authorized users.

## Background

Neuroblastoma (NB), the most frequent childhood extracranial solid tumor, is characterized by heterogeneous clinical and biologic behaviors. Despite aggressive multimodal therapy, the mortality rate for patients with high-risk neuroblastoma (HR-NB) remains greater than 60% [1]. Therefore, exploring common mechanisms underlying heterogeneous patients would aid in developing additional prognostic indicators and combination treatments for patients.

The implications of multigene models in precise medicine remain inexplicable largely due to unclear biological pathways underlying each multigene model. Previous studies have developed multigene models from transcriptomic profiles that were prognostic for HR-NB [2, 3]. These multigene models are cohort- and parameter-dependent since they were derived through a supervised machine-learning method. Such dependence is impracticable because researchers have to normalize the profiles of a large-sized cohort before a prediction, meaning estimates can change when adding additional samples. We recently proposed an approach [4, 5] that analyzes relative expression levels of gene-set pairs (RXA-GSP), which can derive a personalized prognostic index from gene expression profiles. Nevertheless, this method requires two groups of predefined gene candidates: one group marks favorable outcomes and the other group marks unfavorable outcomes. Therefore, a critical step towards innovative NB risk stratification using RXA-GSP is to not only reveal novel biomarkers but also understand their underlying functional genomics.

It is increasingly accepted that cancer cells show behavior reminiscent of the biological mechanisms ascribed to normal embryonic stem cells (ESCs) (reviewed in [6]). An ESC-associated prognostic expression pattern, the high-expression of transcription factor c-Myc/MAX co-targets combined with the low-expression of Polycomb repressive complex 2 (PRC2)-silencing genes, has been found in multiple types of poorly differentiated tumors particularly adult tumors [7]. In neuroblastoma, both MYC and PRC2 play critical roles. On the one hand, we recently presented a subnetwork of Myc family gene *c-Myc* enriched for genes previously reported as ESC-like cancer signatures by a network analysis of transcriptome data [8]. The other MYC family gene *MYCN* is ESC-functionally essential and sufficient to produce tumors in mouse and zebrafish models [9, 10] and all patients with amplification of the MYCN oncogene are considered high-risk [11, 12]. High-risk patients, even with normal MYCN copy numbers, frequently overexpress targets of Myc family genes [13, 14]. Furthermore, therapeutic targeting of the MYCN or c-MYC signal has been proposed for HR-NB treatment [15, 16]. On the other hand, reactivation of PRC2 targeted tumor suppressors has been proposed for HR-NB [17]. Furthermore, an ESC-like signature was derived from multiple aggressive tumors consisting of both a PRC2 module and a MYC module [18]. Collectively, these previous work suggest an ESC-like mechanism underlying the tumorigenesis of HR-NB.

We hypothesize that the imbalance between MYC-driven oncogenesis and PRC2-induced repression determines, at least in part, the poor prognostic phenotypes shared by heterogeneous HR-NB tumors. A critical sub network underlying this systematical imbalance is frequently disturbed by polymorphisms or somatic mutations and by transcriptional dysregulation, thus can be retrieved from “-omic” landscapes. Advances in high-throughput sequencing have provided an unprecedented opportunity to interrogate genome, transcriptome and functional genomics systematically and facilitate this knowledge discovery. Therefore, to test this hypothesis, this study designs a systems bioinformatics analysis of multiple genome-scale datasets; and characterizes therapeutic candidates by comparing high-risk tumor cells with their differentiation-induced cells, the control components. Retinoic acid (RA) induces HR-NB cell growth and differentiation and thus reverses malignant growth in vitro and in vivo [19–21], therefore RA is used to induce cell differentiation.

## Results

### Identifying prognostic gene-set pairs (GSPs) from significant concurrence of genes showing MYCN-associated expression and exons with verified variants in HR-NB (Fig. 1)

**Fig. 1:**
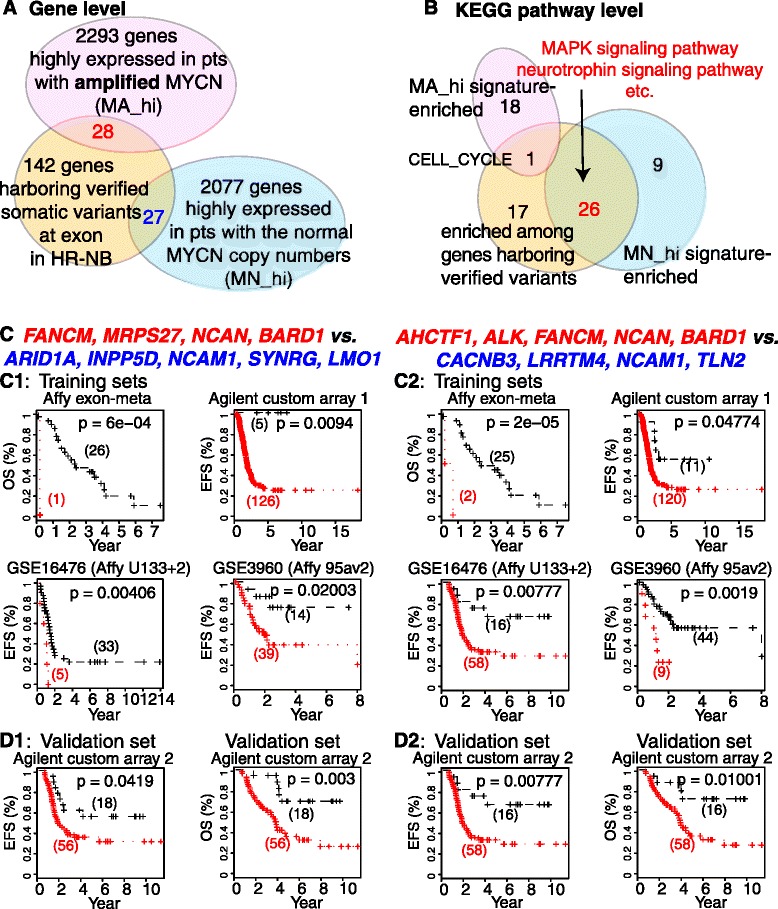
The connection between prognosis, genomic variation, and transcriptional MYCN-association in HR-NB. **a** Significant overlap (*p* = 0.02, OR = 1.5) exists between the 197 genes harboring verified variants at exon and the 4425 MYCN-associated genes. The latter includes those highly expressed in MYCN-amplified patients or in patients with the normal MYCN copy numbers. **b** The pathways that are enriched among genes with verified variants at exon over-represent the pathways that are enriched among genes highly expressed in patients with MN HR-NB (OR = 20, *P* = 7e-3), but are depleted for the pathways that are enriched among genes highly expressed in patients with MA HR-NB (OR = 0.16, *P* = 0.048). **c** Kaplan–Meier plots of two identified prognostic GSPs in four independent training cohorts. **d** Kaplan–Meier analysis of the two GSPs in an independent cohort for event free survival (EFS) and overall survival (OS). In panels **c**-**d**, the *red lines* represent patients with positive indicators (ie, the median expression of the identified genes in *red* is higher than that of the genes in *blue*), and the *black lines* represent patients with negative indicators

Given the genetic heterogeneity of HR-NB, we investigated the concurrence of somatic mutation and transcriptomic dysregulation in patients, in which the needs for genomic and translational advances are both pressing. From published data sets (Additional file 1: Table S1), we identified 4425 genes termed “MYCN-associated” that were significantly up-regulated in MA patients (MA_hi) or MN patients (MN_hi), respectively (Stoffer meta-analysis FDR < 0.001). Comparing these 4425 MYCN-associated genes with a collection of 197 unique genes harboring verified exonic variants (Additional file 1: Table S2) resulted in 55 (28%) genes in common, including 28 MA_hi and 27 MN_hi genes (Fig. 1a). We performed the Fisher’s exact test (FET) using a background of around 21 k human protein-coding genes. An over-representation of genomic mutations among the MYCN-associated genes (*P* < 0.02, odds ratio = 1.4) suggests a common downstream effect on HR-NB tumorigenesis existing among sporadic variation and usually-observed MYCN-associated dysregulation.

We thus hypothesized that diverse genetic and transcriptomic disturbances can lead to a critical signaling pathway dysregulation that underlies tumorigenesis in multiple HR-NB subtypes. To reveal this potential functional commonality, we performed pathway enrichment analysis for these gene signatures (Fig. 1b), using KEGG pathways collected by the MSigdb database (v5.1) [22]. Only one KEGG pathway—the cell cycle pathway—was significantly enriched among both the genes with verified mutation and the MA_hi genes (enrichment criterial: FET FDR < 0.01, odds ratios > 2). In contrast, somatic mutated genes significantly overlapped with MN_hi genes at the pathway level (26 common pathways, OR = 20, *P* = 7e-3). Of these 26 commonly enriched pathways, the MAPK signaling pathway and the neurotrophin signaling pathway have been reported to play roles in regulating the malignant transformation of neuroblasts to neuroblastoma cells [23, 24]. Notably, one third of these 26 commonly enriched pathways—for example, the non-small cell lung cancer pathway (Additional file 1: Table S3)—were cancer-specific pathways. These data suggest that diverse genetic and transcriptomic disturbances may lead to a critical signaling pathway dysregulation underlying tumorigenesis shared among multiple cancer types.

From thousands of genes with MYCN-associated dysregulation, the observed over-representation of genomic variation suggested a way for us to prune the critical signaling pathway dysregulations that could be triggered by diverse genetic or transcriptomic disturbances. From 55 *MYCN*-associated genes that harbor verified genomic mutations in HR-NB, we focused on the 21 best candidate genes that harbor recurrent, verified missense variants (Table 1). Two gene groups were remarkable: the first was ten MA_hi genes (*AHCTF1, ALK, ATM, FANCM, MRPS27, MSH2, MYCN, NCAN, STAG1,* and *BARD1*), and the second was eleven MN_hi genes (*ARID1A, CACNB3, IL16, INPP5D, LRRTM4, MLL5, NCAM1, NRAS, SYNRG, TLN2,* and *LMO1*).

**Table 1:** The 21 best candidate genes that are MYCN-dependent and harbor recurrent, verified missense variants in HR-NB

Hugo Symbol	MYCN-associated expression	Case USI	Chromosome	Start_Position	Variant Classification	Mycn statues	Verification Method
AHCTF1	MA-hi	TARGET-30-PAPNEP	1	247,063,482	Missense	amp	
		TARGET-30-PASAZJ	1	247,014,426	Missense	non-amp	(2)
ALK	MA-hi	N130 T	2	29,286,167	Missense	amp	(1)
		N160 T	2	29,286,167	Missense	gain	(1)
		N744 T	2	29,286,167	Missense	non-amp	(1)
		N198 T	2	29,297,200	Missense	non-amp	(1)
		TARGET-30-PAKXDZ	2	29,445,213	Missense	amp	(3)
		TARGET-30-PANRVJ	2	29,443,697	Missense	non-amp	(4)
		TARGET-30-PARKNP	2	29,443,697	Missense	non-amp	(4)
		TARGET-30-PARLTG	2	29,443,696	Missense	non-amp	(4)
		TARGET-30-PARZIP	2	29,443,696	Missense	non-amp	(4)
		TARGET-30-PAPRPR	2	29,443,695	Missense	amp	(4)
		TARGET-30-PASVRU	2	29,443,695	Missense	amp	(4)
		TARGET-30-PATFXV	2	29,443,695	Missense	amp	(4)
		TARGET-30-PALAKE	2	29,443,695	Missense	amp	(3)
		TARGET-30-PANZVU	2	29,443,695	Missense	non-amp	(3)
		TARGET-30-PATFCY	2	29,436,875	Missense	non-amp	(4)
		TARGET-30-PAINLN	2	29,436,860	Missense	non-amp	(3)
		TARGET-30-PASKFV	2	29,432,664	Missense	amp	(4)
		TARGET-30-PANWRR	2	29,432,664	Missense	non-amp	(4)
		TARGET-30-PANXJL	2	29,432,664	Missense	non-amp	(4)
		TARGET-30-PAPTFZ	2	29,432,664	Missense	amp	(4)
		TARGET-30-PAPTLV	2	29,432,664	Missense	amp	(4)
		TARGET-30-PARURB	2	29,432,664	Missense	non-amp	(4)
		TARGET-30-PALNLU	2	29,432,664	Missense	non-amp	(3)
		TARGET-30-PANBCI	2	29,432,664	Missense	amp	(3)
		TARGET-30-PASAZJ	2	29,432,664	Missense	non-amp	(2) (4)
		TARGET-30-PAREGK	2	29,443,695	Missense	non-amp	(2)(5)
ATM	MA-hi	TARGET-30-PANYGR	11	108,196,934	Silent	non-amp	
		TARGET-30-PANRHJ	11	108,200,975	Missense	non-amp	
		TARGET-30-PANRRW	11	108,196,798	Missense	non-amp	(2)
FANCM	MA-hi	TARGET-30-PASXRG	14	45,646,146	Missense	amp	(4)
		TARGET-30-PARHUX	14	45,657,091	Splice_Site	amp	(4)
		TARGET-30-PARETE	14	45,658,176	Nonsense	amp	(4)
		N492 T	14		Mut. Splice Junction	non-amp	
MRPS27	MA-hi	TARGET-30-PAPSKM	5	71,519,665	Missense	non-amp	(2)(5)
		TARGET-30-PAPSKM	5	71,522,027	Splice_Site_GWAS SNP	non-amp	(2)(5)
MSH2	MA-hi	TARGET-30-PAITCI	2	47,641,416	Silent	non-amp	
		TARGET-30-PARKNP	2	47,693,815	Frame_Shift_Del	non-amp	(4)
		N538 T	2	2,367,537	Protein Fusion	amp	
MYCN	MA-hi	TARGET-30-PAPLSD	2	16,082,317	Missense	non-amp	(4)
		TARGET-30-PARCRR	2	16,082,317	Missense	non-amp	(4)
		TARGET-30-PARVNT	2	16,082,317	Missense	non-amp	(4)
		TARGET-30-PASLGS	2	16,082,317	Missense	non-amp	(2)(3)(5)
		NB-1744-Tumor	19	19,337,844	Missense		(4)
		N619 T	2	16,003,271	Missense	amp	(1)
NCAN	MA-hi	TARGET-30-PANIPC	19	19,337,844	Missense	amp	(4)
		TARGET-30-PAPKXS	19	19,338,389	Missense	non-amp	(4)
		TARGET-30-PANLET	19	19,359,524	Missense	non-amp	(4)
STAG1	MA-hi	TARGET-30-PAIPGU	3	136,117,607	Missense	non-amp	
		TARGET-30-PARDCK	3	136,078,044	Missense	non-amp	
		TARGET-30-PAPZFW	3	136,057,265	Frame_Shift_Del	non-amp	(4)
BARD1	MA-hi	TARGET-30-PATGWT	2	215,595,215	Nonsense; 1p-;17q+	amp	(3)
		TARGET-30-PAHYWC	2	215,657,051	Nonsense; 9p-; 10q-;17q+	amp	(3)
		GWAS	2	215,672,546	GWAS SNP		(6)
		GWAS	2	215,635,794	GWAS SNP		(6)
ARID1A	MN-hi	TARGET-30-PALNLU	1	27,099,000	Missense	non-amp	(3)
		TARGET-30-PALXHW	1	27,106,214	Missense	non-amp	(3)
		N554 T	1	26,970,220	DEL	amp	(1)
CACNB3	MN-hi	TARGET-30-PAMDAL	12	49,220,231	Missense	amp	(4)
		N608 T	12	47,505,015	Missense	amp	(1)
IL16	MN-hi	TARGET-30-PAIMDT	15	81,561,966	Missense	non-amp	(3)
		N170 T	15	79,385,873	Missense	non-amp	(1)
INPP5D	MN-hi	TARGET-30-PANUIF	2	234,070,424	Missense	non-amp	(4)
		TARGET-30-PANRVJ	2	234,098,527	Missense	non-amp	(4)
LRRTM4	MN-hi	TARGET-30-PATEPF	2	77,746,658	Frame_Shift_Ins	non-amp	(4)
		N717 T	2	77,600,317	GWAS SNP	amp	(1)
MLL5	MN-hi	TARGET-30-PARBLH	7	104,748,301	Missense	non-amp	
		TARGET-30-PANYGR	7	104,753,479	Missense	non-amp	(2)
		N198 T	7	104,437,581–104,447,802	Deletion	non-amp	
NCAM1	MN-hi	TARGET-30-PAPZFW	11	113,130,966	Silent	non-amp	(4)
		TARGET-30-PASGUT	11	113,077,981	Splice_Site	non-amp	(4)
NRAS	MN-hi	TARGET-30-PANBSP	1	115,258,745	Missense	non-amp	(4)
		TARGET-30-PAPTMM	1	115,256,530	Missense	amp	(2)(5)
SYNRG	MN-hi	TARGET-30-PARDIW	17	35,930,941	Missense	non-amp	
		TARGET-30-PASAZJ	17	35,956,340	Missense	non-amp	(2)(5)
TLN2	MN-hi	TARGET-30-PASRFS	15	63,029,117	Silent	non-amp	
		TARGET-30-PARNNG	15	63,030,490	Missense	non-amp	
		TARGET-30-PARIRD	15	62,942,372	Missense	amp	(2)(5)
LMO1	MN-hi	GWAS	10	8,252,853	GWAS SNP		(6)
		GWAS	10	8,238,639	GWAS SNP		(6)

The information was collected from the Table S3 of Pugh 2013 Nature paper and the Tables S1, S3, S6 of Molenaar 2012 Nature paper. All patients are with high-risk neuroblastoma (TARGET_NBL_clinicalCovariates20120702.txt)

Validation Method: (1) linkage disequilibrium analysis; (2) PCR/HiSeq; (3) Sequenom; (4) PCR/MiSeq; (5) PCR/Sanger; (6) GWAS

We hypothesized that a unifying prognostic signature exists among HR-NB tumors regardless of the MYCN status. To derive this signature, we applied our previously published RXA-GSP approach [4, 5] to these two gene-groups. We identified two unifying prognostic gene-set pairs (GSPs) from these 21 best candidate genes using a collection of 251 HR-NB samples (Cox regression *p* < 0.05 for event-free survival in all four training cohorts and Liptak joint FDR <0.05) (Fig. 1c). Figure 1d illustrates the evaluation of prognostic significance in an independent cohort (theoretical *P* = 0.042, 0.0078 for event-free survival, and empirical *P* = 0.115 and 0.027, respectively). These two GSPs also predicted overall survival significantly (theoretical *P* = 0.003, 0.001 as shown in Fig. 1e and empirical *P* = 0.027 and 0.015, respectively). Note that the somatic mutations of 12 (57%) candidates occurred in both MA and MN patients. However, eight (38%) genes harbor the recurrent somatic mutations of only MN patients while one gene (5%) harbors the recurrent mutations of only MA patients, suggesting a preference of genomic variants in patients carrying the normal *MYCN* copy numbers.

### Functional enrichment and network analysis link the HR-NB prognostic GSPs with two components of an ESC-like cancer signature (Fig. 2)

**Fig. 2:**
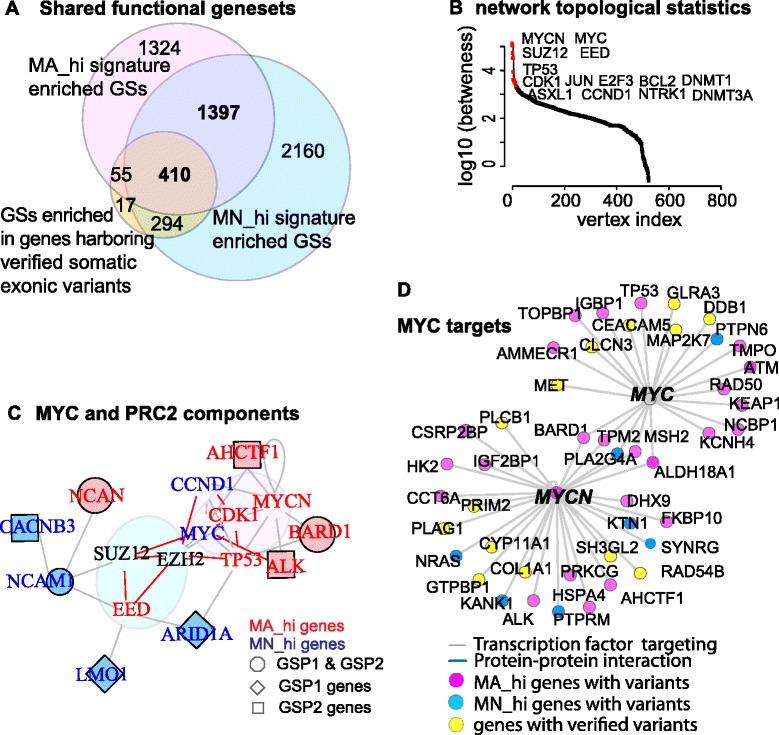
Functional enrichment and network analysis link the HR-NB prognostic signatures with the MCY and PRC2 components of an ESC-like gene signature. **a** Among the three multigene signatures, we evaluated the over-representation of all six types of functional gene-sets downloaded from MSigDB v3.1. The enriched gene-sets (FDR < 0.01, GS size < 2000) show significant concurrence of MA_hi genes and MN_hi genes (*p* < 2e-16, odds ratio > 8, the *pink* and *blue circles*), regardless of their non-overlap on the gene-level. Additionally, these gene-sets significantly co-occur with the gene-sets what were enriched among genes harboring verified variants at exon (*p* < 2e-16, odd ratios > 100, the saffron circle and the union of the other two circles). **b** Rank the 4460 HR-NB associated proteins according to their topological betweenness in the PPI network. **c** A model of imbalanced ESC-like signatures with PRC2-targets and MYC/MAX-targets at the transcript and protein-protein-interaction (PPI) levels. Weinberg’s group reported that this human ESC-like signature was associated with histologically poorly differentiated tumors (breast, glioma, and bladder cancers). PPI (STRING v9.05) reveals a critical role of *MYCN*, *TP53* and *NCAM1* in tuning the prognostic imbalance. Distinct colors code genes for genomic variants, showing transcriptional *MYCN*-association, or both. We highlight the genes of the identified prognostic GSPs with a larger node-size in the network.﻿ ﻿**d**﻿ MYC proteins preferentially target MA_hi genes. Network-node colors decode genes with genomic exonic variants, genes showing transcriptional MYCN-association, or both. TF: transcript factor

To understand the biology underlying the identified prognostic GSPs, we performed functional gene-set enrichment analysis (MSigDB v3.1, Fig. 2a). Among the HR-NB associated genes (including the two prognostic GSPs, the MA_hi and MN_hi genes, and the genes with somatic mutations), we discovered eight commonly enriched transcription regulators and a cancer module previously described in multiple cancer types (*KRAS* **,** *EED*, *HFH3, SMAD4* and PRC2 components for MN_hi whereas *MYCN, MYC* and *E2F4* for MA_hi genes, FDR < 0.01, odd ratio = 2). These eight regulatory models indicated critical upstream regulators of tumorigenesis in heterogeneous HR-NB. For example, the first GSP was enriched for tumor suppressors (eg, the targets of PRC2 component EED given that PRC2 has been previously reported to repress tumor suppressors [17]) whereas the second GSP was enriched for targets of oncogenes (such as *FGFR1*, *MYCN*, genes in the neurite outgrowth pathway, and common cancer module #1 [25]). In consistent, the MN_hi signature exhibits a relatively favorable prognostic component (tumor suppressor *SMAD4*-induced targets (*p* < 0.0009) [26] and oncogenic *KRAS* suppressed targets (*p* < 0.0006) [27]) while the MA_hi signature denotes an unfavorable prognostic component (oncogenic *E2F4* induced targets [28] (*p* < 1e-8)). These observations together with the preference of genomic variants in MN_hi signature genes suggest that loss of tumor suppressing function is a major role of genomic variation.

We next aimed to further reveal the regulatory mechanisms underlying these eight regulatory models in HR-NB (Fig. 2b), or more broadly, other aggressive tumors where similar common pathways contribute to oncogenesis. To this end, we further performed protein-protein-interaction (PPI) analysis. We collected 4460 genes, including not only the ‘HR-NB associated genes’ (differentially expressed and the somatic mutated ones) but also the targets of the eight regulators (Additional file 1 **:** Table S4) that were identified by a gene-set enrichment analysis of these HR-NB associated genes. These regulators were: MYCN or Myc/Max complex, three core components of PRC2 (EED, EZH2, and SUZ12), and the other identified transcription regulators (FGFR1, KRAS, E2F4, HFH3, SMAD4). We hereafter name this PPI subnetwork as ‘HR-NB associated PPI’.

In the HR-NB associated PPI, topological bottlenecks (network nodes with high betweenness that could greatly influence information flows) highlighted the potentially essential network nodes. A PPI bottleneck analysis pinpointed both MYC protein family (*NMYC* and *MYC*) and the core PRC2 components (SUZ12, EED) (Fig. 2b), consist with a published ESC-like signature derived from multiple types of aggressive tumors [18]. Given that we have observed that diverse genomic and transcriptomic disturbances dysregulated the same signaling pathway and MYC oncogenes frequently played as critical regulators, we concluded that a linkage exists between the two HR-NB prognostic GSPs and the ESC-like cancer signature in HR-NB.

To understand the potential underlying mechanism of ESC-like signature in the identified GSPs, we induced the HR-NB associated PPI by both GSP markers and network bottlenecks. We observed that histologically poorly differentiated tumors show preferential overexpression of MYC-targeted genes combined with repression of PRC2-targeted genes [7]. On the one hand, we identified four *PRC2*-silencing targets (*ARID1A, CACNB3, NCAM1, LMO1*) being as MN_hi (up-regulated in relatively favorable outcome MN patients), which were previously found to be repressed by PRC2 components [7] (Fig. 2c, blue nodes). Three out of these four MN_hi genes in the first GSP significantly over-represent *EED* targets in human ESCs (FET *p* = 0.00014). As expected, *EED*, which is required for maintenance of the self-renewal in mouse ESCs [29, 30], was more highly expressed in MN samples than in MA samples (fold changes > 1.35 in both platforms, Stouffer FDR < e-10). Two MN_hi genes within the second prognostic GSP, *NCAM1* and *CACNB3,* were targets of epigenetic repressor H3k27me3 that preferentially associated with PRC2 (FET *p* = 0.0064) [7, 31]. On the other hand, the c-Myc/Max complex [7] preferentially targeted the MA_hi genes (Fig. 2d, red nodes). MYCN/c-MYC are important as they directly regulate TP53 which is a pro-apoptotic gene overexpressed in HR-NB and are independent of other markers [13]. Collectively, these results indicate that deregulation of several key regulators systematically tunes the imbalance of unfavorable and favorable features, including tumor-suppressors *TP53, ATM* and *SMAD4*, oncogenes *KRAS* and *E2F4*, and genes with both potentials (*MYCN* and *E2F4* [32, 33]).

### Both MYC-binding targets and PRC2-silencing targets over-represent cell differentiation markers, but PRC2 repressed targets are preferentially mutated in HR-NB (Fig. 3)

**Fig. 3:**
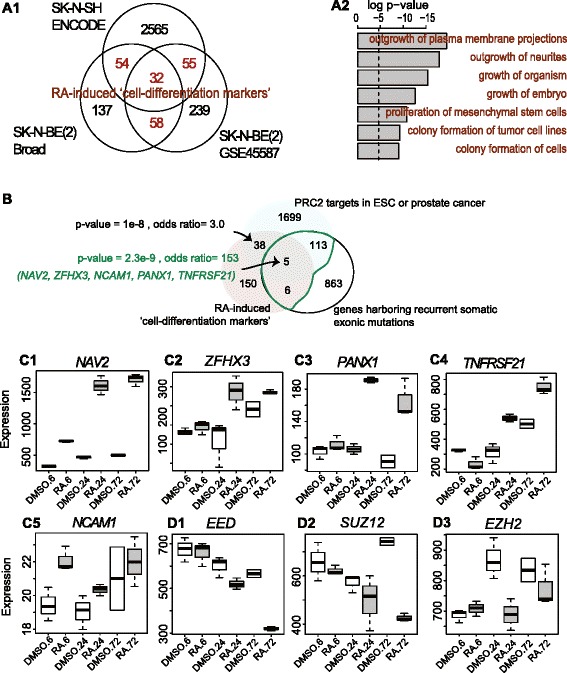
RA-induced cell-differentiation markers in HR-NB over-represent the PRC2-silencing targets and somatic mutations. **a** Identification and functional enrichment analysis of RA-dependently expressed genes. Sub-panel 1 is Venn diagrams demonstrating how to identify the core set of RA-dependent differentiation markers. Sub-panel 2 is a bar plot of the Fisher’s exact test results for the enriched functions among the RA-induced biomarkers (Ingenuity pathway analysis). **b** Venn diagram of the targets of PRC2, RA-induced genes, and genes harboring recurrent somatic mutations in HR-NB. Significance of enrichment is estimated using the Fisher’s exact test against approximately 21,000 human genes. **c** The mRNA levels of the five markers pinpointed in Panel B1 increased after RA-induced cell differentiation. The mRNA levels of the PRC2 components decreased after RA-induced cell differentiation. Box-and-whisker plots represent individual value distributions that are categorized as vehicle (*left*) and RA-treated (*right*) along with treated hours (x-axis) in the SK-N-BS cells (GSE45587). The central box represents the values of an expression (*y-axis*) from the lower to upper quartile. The *middle line* represents the median. The *horizontal line* extends from the minimum to the maximum value within 1.5 times of the interquartile range from the box. **d** The mRNA levels of three genes encoding PRC2 component increased after RA-induced cell differentiation

We hypothesized that HR-NB aggressiveness requires the mediation of not only MYCN but also PRC2 based on the observation that PRC2 preferentially repressed outcome-favorable MN_hi genes. We also showed the concurrent somatic mutations and MN_hi genes in HR-NB. To further understand the effects of PRC2 targeting in HR-NB, we used Retinoic Acid (RA) as a study model of HR-NB cell differentiation [20, 21]. We identified a core set of 199 “cell-differentiation markers” (Fig. 3A1) that were consistently RA-inducible from two out of three published transcriptomic datasets using a RA-sensitive HR-NB cell line SK-N-BE(2) [34, 35] or SK-N-SH (ENCODE). These 199 RA-induced cell-differentiation markers significantly over-represented a function of cellular growth and proliferation (97 involved genes, overall *p* < 2.0e-4, IPA analysis, Fig. 3A2).

We further asked the question whether there is a measurable relationship among the identified prognosis, cell differentiation, and ESC-like signatures. We evaluated enrichment among these gene signatures and found that loss of PRC2 inhibition interrupts tumor cell differentiation in HR-NB. The cell-differentiation markers in HR-NB remarkably over-represented not only the 2104 outcome-favorable MN_hi genes (71 overlap, *p* = 4.8e-16, odds ratio = 3.6) but also the PRC2 core targets in ESCs [7] or cancer (Fig. 3b). We identified 43 cell-differentiation markers targeted by PRC2 (either showing *EZH2*-repression in prostate cancer or PRC2-silencing in ESC), presenting a significant enrichment among RA-induced genes and PRC2-silencing targets (*p* = 1e-8, odds ratio = 3). Such enrichment increases if we concentrate on the subsets of genes harboring recurrent somatic mutations (odd ratios = 153, Fig. 3b), which suggests that these gene mutations are critical to PRC2 function. Additional enrichments existed between the PRC2 targets and the outcome-favorable MN_hi genes harboring recurrent somatic variants (*p* = 9.3e-5, odds ratio = 2.6), and between the RA-induced cell-differentiation markers and the MN_hi genes harboring recurrent somatic variants (*p* = 3.2e-8, odds ratio = 89). Specifically, five RA-inducible genes (*ZFHX3, NAV2, NCAM1*, *PANX1*, and *TNFRSF21*) were the PRC2 targets that harbor recurrent somatic mutations. Published time-course experiments verified the RA-inducible feature for these five markers (Fig. 3c) which are anti-correlated with the expression levels of PRC2 coding genes (Fig. 3d)﻿.

As a control, we identified a set of 156 “cell-dedifferentiation markers**”** that were consistently RA-repressed (Additional file 2: Figure S1A). These 156 “cell-dedifferentiation markers” significantly over-represented the 775 MYC/MAX targets identified by ChIP-on-ChIP [7] (21 overlap, *p* = 3.1e-7, odds ratio = 4.1, Additional file 2: Figure S1B). Interestingly, no recurrent somatic mutation was reported for these cell-dedifferentiation markers. We conclude that a high rate of the mutated differentiation markers over the mutated dedifferentiation markers may be associated with aggressiveness in HR-NB.

### Inhibiting PRC2 decreased cell growth after increased the expression of ZFHX3 in HR-NB cells (Fig. 4)

**Fig. 4:**
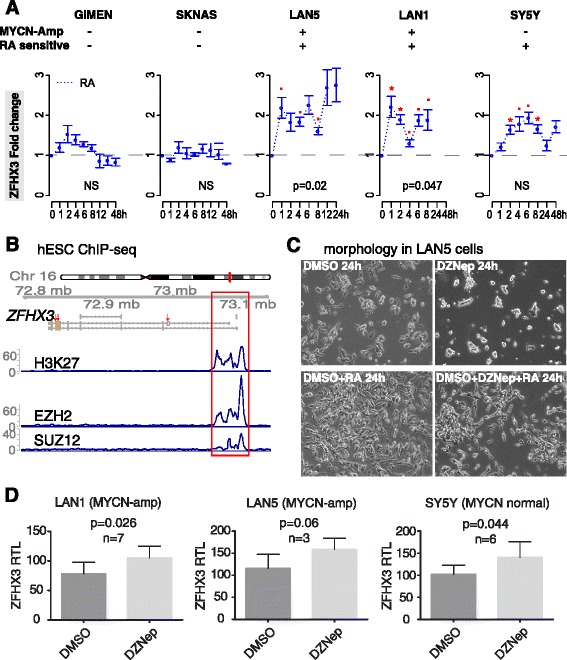
Inhibiting PRC2 component affects cell morphology and increases the mRNA expression of cell differentiation marker ZHFX3. **a** ZFHX3 mRNA expression increases after RA-treatment in three RA-sensitive cells but not the two RA-insensitive cells. The MYCN amplification status and the RA-sensitive or resistant states of five human NB cell lines are given at the top of this panel. X-axis gives the RA-treatment hours, and Y-axis presents the fold change (2^-ddCt) of relative transcript level using RT-PCR at each time point. Data represent mean values ±95% confidence intervals on the estimates of the means from 2 to 6 biological replicates, each with two technical repeats. The significance for fold-change after RA-treatment without blocking PRC2 using the one-tailed Student t-test is represented as: (0.05 ≤ *p* < 0.1), * (0.01 ≤ *p* < 0.05), ** (0.001 ≤ *p* < 0.01). **b** Genomic view (hg19) of the gene ZFHX3. Three red arrows point reported somatic mutations in HR-NB. The *red box* highlights a PRC2 occupancy at ZFHX3 promoter in human ESC cells (ENCODE data). **c** Cell morphology was examined in the LAN5 cell line after 24 h of treatment with vehicle (A1), DZNep (A2), RA (A3), or DZNep and RA (A4), respectively. Pictures were taken using the 20-fold magnification of a Leica DM IRB light microscope. **d** The *ZFHX3* mRNA levels were increased after 8 h of DZNep treatment. X-axis gives the three HR-NB cell lines, and Y-axis presents the DMSO-normalized relative transcript level (RTL). Real-time PCR was employed to examine quantitative differences in mRNA expression between dimethyl sulfoxide (DMSO) and DZNep-treated cells. These relative expression levels (fold change (2^-ddCt)) were initially normalized to the mean of Glyceraldehyde 3-phosphate dehydrogenase (GAPDH). Data shown are mean relative expression levels ± standard deviation of experiments. The *p*-value of a two-tailed t-test is given in each scenario, followed by the number of biological replicates

Of the five PRC2-targeted cell-differentiation markers with somatic mutations in HR-NB, we focused on the transcription factor *ZFHX3* (zinc finger homeobox 3). ZFHX3, also known as AT motif binding factor 1 (ATBF1), has been reported to induce differentiation and cell cycle arrest in neuronal cells [36]. To confirm that ZFHX3 marks HR-NB cell differentiation, we carried out real-time quantitative PCR analysis in cells with and without RA treatment (Fig. 4a). The mRNA levels of *ZFHX3* were consistently increased and significantly increased in three RA-sensitive NB cell lines (SY5Y, LAN1, LAN5) after RA-treatment, but remained unchanged within 24 h in two RA resistant cell lines (GIMEN, SKNAS). This data confirms that *ZFHX3* is a cell differentiation marker in RA-sensitive HR-NB cells.

We assumed that PRC2 repression plays a role in blocking NB differentiation and inducing proliferation, which is partly measurable through ZFHX3. This hypothesis was based on the three observations: 1) ZFHX3 was targeted by PRC2 in ESC cells. 2) Reduced or absent *ZFHX3* expression characterized extremely malignancy in neuroblastoma and other solid tumors with a high frequency of metastasis [37–41]. 3) The PRC2 complex proteins intensively occupy ZFHX3 promoter in human ESCs (Fig. 4b).

To test the hypothesis that PRC2 impacts the pathway signaling of cell differentiation and tumor growth, we treated HR-NB cells with DZNep (depsipeptide, 3-deazaneplanocin A - an inhibitor of cellular methyltransferase that reactivates PRC2-repressed genes in neuroblastoma) by dissociating the PRC2 complex [17, 42] (Fig. 4c). The RA-sensitive LAN5 cells were pretreated for 24 h with vehicle (DMSO) or DZNep in vehicle. In terms of cell morphology, we found a decreased percentage of cell survival after treating cells with DZNep for 24 h and an increased percentage of cell survival after treating cells with RA for 24 h. Interestingly, we observed a stronger cell apoptosis induced by DZNep treatment than a cell differentiation induced by RA treatment in the LAN5 cells (Fig. 4c). Thus, the pharmacological inhibiting PRC2 in RA-treated SH-SY5Y cells may antagonist the role of RA in cell differentiation. This result serves as a functional validation that PRC2 pharmacological inhibition with DZNep increases tumor cell apoptosis which we and others have observed in HR-NB and colon cancer [43].

To quantitatively evaluate the impact of PRC2 on its targeted cell-differentiation marker ZFHX3, we measured the expression changes after inhibiting PRC2 using DZNep (Fig. 4d). In the early DZNep treatment stage (8 h) in three RA-sensitive HR-NB cell lines, *ZFHX3* mRNA expression increased significantly (*P* = 0.026, 0.06, and 0.044, respectively). Collectively, our data suggests that PRC2 histone methyltransferase activity may constitute a new epigenetic therapeutic strategy to mediate tumor cell growth and differentiation in HR-NB by rescuing PRC2-silencing.

## Discussion

Cancer cells have deviated from the normal genome by acquiring and selecting a set of mutations that enable their malignancy [44]. These changes can be germline variations, somatic mutations, or upstream regulators that trigger the cancer process. By assembling and cross-analyzing genomic variants, gene expression changes, prior knowledge, clinical outcomes, and using the RA-induced cell differentiation model, we modeled HR-NB aggressiveness beyond prognostic multigene signatures. One knowledge gap we have addressed here is that previous prognostic gene signatures lack overlap. As a solution, our model uses an individualized index of relative expression between poor- and good-outcome markers. Our results indicate the existence of unified prognostic signature in HR-NB that is MYCN-amplification independent. For example, *NCAM1* (also known as *CD56*) is a main carrier for the neural crest stem cell marker and its reduced expression is correlated with unfavorable prognosis in neuroblastoma with distant metastases, regardless of the *MYCN* amplification status [45, 46].

The two identified prognostic gene-set pairs for individual patients pinpointed essential HR-NB biomarkers. Among the six relatively poor prognostic markers, ALK and BARD1 have been reported as neuroblastoma predisposition genes [47, 48]. The gain-of-function mutation in ALK acts synergistically with MYCN to drive NB development and indicates worse event-free survival [49, 50]. BARD1β not only presents the characteristics of neuroblastoma oncogenes but also enhances MYCN-stabilized high-risk phenotypes [48]. These reports confirm the unfavorable prognostic effects of ALK and BARD1 alongside the MYCN amplification in the two gene-set pairs. Among the eight relatively good prognostic markers, the neuroblastoma oncogenic roles of the highly-expressed LMO1 and the deletion of ARID1A have been evaluated [51, 52]. On the other side, somatic copy number gains of LMO1 were significantly correlated with MYCN none-amplification, consistent with its MN_hi classification [52]. Tumor suppressor potential of ARID1A has been reported [53], in accordance with the relatively favorable prognostic roles of the MN_hi signature. What stands specifically intriguing is the MYCN-independent prognosis of the relative expression of these markers in individuals. This finding is important as among high-risk population none of the established risk markers including the MYCN copy number can efficiently stratify outcomes [54].

Of particular interest is the PRC2 targets anchored with the ESC-like features which are measurable by RA-induced cell differentiation. Genes in the two identified prognostic multigene signatures added a new pediatric cancer type, neuroblastoma, to the impacts of the cancer prognostic ESC-like signature showing preferential high-expression of MYC targets combined with low-expression of Polycomb-regulated genes [7]. We identified five PRC2-repressed cell-differentiation markers with reported somatic mutations (*ZFHX3, NAV2, NCAM1*, *PANX1*, and *TNFRSF21*) in HR-NB. Among them, we found that the mRNA expression of *ZFHX3* is RA-inducible in HR-NB, which agrees with studies on *ZFHX3* in normal cerebellar neurons [21, 55, 56]. We further validated that blocking PRC2 using the inhibitor DZNep increased the mRNA expression of *ZFHX3*. These data suggest that PRC2-silencing plays an important role in tumor cell differentiation that can be measured by *ZFHX3*. Mutation of ZFHX3 has been reported for HR-NB and autism [37, 57], however, its functional impacts remain unclear. One previous research study proposed ZFHX3 as a tumor suppressor in non-small cell lung cancer [58]. We propose ZFHX3 to be a new tumor suppressor candidate in HR-NB, which is worth further investigation.

Innovatively**—**in terms of cancer biology, our results highlight MYC and PRC2 as stem-cell-associated, tumor-specific regulators [59, 60] in pediatric HR-NB. Recent study suggested that MYC proteins maintain stem cell identity through recruiting PRC2 repression in mouse ESCs [61]. A deeper understanding about the cross-talk between the MYC proteins and PRC2 components represented in ESC-like signature, and how does an imbalance in this cross-talk lead to malignancy, would shed novel light on a reduced relapse and mortality rate of unfavorable cancers.

Approaching systematic modeling from the computational aspect, instead of focusing on individual aberrations, multiple genomic scales enables a holistic view of how multiple aberrations alter one signaling network within high-risk tumor cells [62, 63]. These models, however, need to be integrated in an iterative way wherein predictions that arise from informatics are constrained by experimental evaluation. This study demonstrates the benefit of integrating systems bioinformatics with cancer researches. Two robust mythologies can be applied to other aggressive tumors for biomarker discovery. First, the novel multi-scaled functional enrichment and network analysis established a link between the prognostic transcription, the somatic mutation with low frequency (noisy observations), and the ESC-like gene signature (priori knowledge). This linkage was confirmed by in vitro experiments in HR-NB cell lines. Second, an index of prognosis was designed for individualized expression profiling, which helps build new biological hypotheses.

## Conclusion

This work demonstrates a hypothesis-driven incorporation of genomic landscapes and transcriptomic profiles into a knowledge-based precision prognosis and biomarker discovery. This systematics approach can be applied to more biological hypotheses, and even broadly to other aggressive tumor studies.

## Methods

### Data

#### Transcriptome

We downloaded gene expression profiles of 488 patients with HR-NB and their clinical information from GEO [64] or ArrayExpress [65] (Additional file 1: Table S1). Additionally, we collected gene expression data pertaining to RA-treatment in HR-NB from the Broad Institute [34] and GEO (GSE45587 [35]) for a cell line SK-N-BE(2) and from ENCODE for another cell line SK-N-SH.

#### Exonic variants

We collected verified, somatic missense-variants at exon from three resources: those identified from TARGET (the National Cancer Institute’s Therapeutically Applicable Research to Generate Effective Treatments, tier 1 data, *n* = 240) [37], supplementary materials in two recent whole-genome sequencing studies [37, 53, 66], and recurrent germline genomic variants that predispose individuals to HR-NB from GWAS [52, 67]. For simplicity, we refer to both types of variants as “genomic variants” hereafter. The collection resulted in a set of 987 genes harboring HR-NB-associated, recurrent variants. From which, the genomic variants of 197 genes have been validated using mass-spectrometric genotyping, PCR-based re-sequencing [37] and an additional SNP array [67], or linkage disequilibrium analysis [66] (Additional file 1: Table S2).

#### *MYC and PRC2* target genes

*PRC2* targets were identified by ChIP-on-chip in human ESCs for the polycomb proteins *H3K27me3, SUZ12,* and *EED* [7, 31]. We additionally collected ChIP-seq data from ENCODE [68] for PRC2-binding (H3K27me3, SUZ12, and EED) in human ESC cells.

### Identification of MYCN-associated gene signature via meta-analysis of expression profiles

We compared the transcriptomic profiles of patients with *MYCN*-amplification (>ten copies, MA) to those with the normal copy numbers of *MYCN* (2 copies, MN) in a collection of 556 primary patients from four independent studies (Additional file 1: Table S1). To increase the statistical power, we pooled samples measured on the same platform into a large dataset after normalization [69] and then applied an empirical Bayes approach [70] to shrink the batch effect [71]. We performed differential expression meta-analysis as previously described [72]. Under a false discovery rate (FDR) less than 0.001, we identified 2321 and 2104 *MYCN*-associated genes significantly up-regulated in MA patients (MA_hi) and MN patients (MN_hi), respectively. The expression levels of these genes exhibited cross-platform consistency on both the gene- level and the exon- level (or were significant on one platform but were not covered by the other platform) after pooling samples and performing meta-analysis [70], thus ensuring the statistical sensitivity.

### Integrating transcriptomic and genomic information

We intersected the genes harboring germline or verified, missense variants at exon with the MYCN-associated signature and found a 55-gene overlap. We evaluated the chance of overlap between gene signatures using the Fisher’s exact test (FET). We further focused on a subset of 21 genes for the downstream analyses, which harbored recurrent mutations in HR-NB and one germline variant that may play more critical roles than those non-recurrent variants.

### Identification of prognostic gene-set pairs (GSPs)

We defined the poor-outcome candidates from the MA_hi genes and the good-outcome candidates from the MN_hi genes, based on the two following observations. First, the MYCN-associated signature covers well-known HR-NB prognostic signatures in HR-NB and the functional MYCN signature (*p* < 0.004). Second, the distribution of coefficients of all 2 million possible GSPs gives more positive (unfavorable) predictions than by chance in a stratified Cox proportional hazards model.

We evaluated all 2,062,468 possible combinations of GSPs and selected the prognostic indicators with the GSPs meeting two following criteria: 1) The GSPs were significant in survival meta-analysis (FDR <5%), ensuring a cohort-independent and array-independent prognosis. 2) The GSPs indicated adverse EFS with a hazards ratio larger than 1 and the survival log-rank tested *p* < 0.05 in each computational cohort.

### Functional enrichment and regulatory network analysis

We examined pathway enrichment on the genes harboring verified genomic variants and the identified MYCN-associated genes using the Bioconductor seq2pathway package [73]. Among 185 KEGG pathways (MSigDb v5.1), those met the criteria of an odds ratio > 2 and a FDR < 0.01 were identified (Fig. 1b). We further evaluated the enrichment for MSigDB (v3.1)-defined functional gene-sets. A HR-NB-associated network was built based on the identified gene signatures and the over-represented upstream transcription factors. Network linkage was generated for any two genes if their protein products interact (STRING v9.05) [74] or exhibit protein-DNA/RNA interactions (MSigDB), using Cytoscape software [75].

### Identifying RA-responsive genes

For the differential expression analysis between the RA-treated and control samples [34, 35], we calculated both static and dynamic statistics using the Bioconductor Limma [76], DEseq2 [77], and edgeR [78] packages. Specifically in the SH-N-BS(2) cells, 281 genes were significantly up-regulated after 5d of RA-treatment compared with the vehicle (ethanol) (limma test, FDR < 0.05, FC > 1.5), and 385 genes were significantly up-regulated during 6-24 h of RA-treatment (generalized linear analysis of variance, FDR < 0.05, FC > 1.5). Additionally, 2724 genes were significantly up-regulated in the RA-treated SK-N-SH cells compared with non-treated cells (FDR < 0.05, FC > 2). Overlaying these three sets, 199 repeatedly RA-induced genes without conflicts are the identified core set (Fig. 3a).

### RA-response experiments using RT-PCR

#### Materials

All trans-RA was bought from Sigma (St Louis, MO, USA, Sigma R2625-50 mg). RA was dissolved in ethanol to make a 5 mM stock. 3-Deazaneplanocin A (DZNep), an EZH2 inhibitor, was purchased from Cayman Chemical (Ann Arbor, MI, USA). DZNep was dissolved in dimethyl sulfoxide (DMSO) and stored at 5 mM concentration.

#### Cells and drug treatment

Human neuroblastoma cell lines were kindly provided by the Cohn Lab at the University of Chicago. We chose five cell lines due to their differing RA sensitivity and genomic characteristics: LAN1, LAN5 are RA-sensitive cell lines [79, 80] exhibiting MYCN amplification, SKNAS and GIMEN are RA-resistant cell lines [80, 81] showing MYCN normality, and SY5Y is an RA-sensitive cell line [80] showing MYCN normality. Cells were cultured in RPMI (Roswell Park Memorial Institute) supplemented with 10% fetal bovine serum and 1% penicillin/streptomycin and maintained at 37 °C with 5% CO2. For drug response, cells were seeded the day before the treatment and dissolved in DMSO. Cells were treated with 5 μM DZNep or reagent (Sigma) for 48 h, and retinoic acid (Sigma) at 10 μM for another 24 h.

#### RT-PCR amplification analyses

We extracted RNA from a maximum of 2–3 million cells per sample using TRIzoL Reagent (Ambion, Invitrogen) according to the manufacturer’s instructions. After RNA extraction, equal amounts of total RNA from different cell lines (1μg) were retro-transcribed using the SuperScript III First-Strand Synthesis System for RT-PCR (Invitrogen, Carlsbad, CA, USA) in the conditions described by the manufacturer. The primer sequences used for RT-PCR amplifications were given in Table 2.

**Table 2:** RT-PCR used primer sequences

Gene	Primer sequences used for amplifications
GAPDH-F	5′ GGAGTCCACTGGCGTCTTC 3′
GAPDH-R	5’ATCTTGAGGCTGTTGTCATACTTC3’
ZFHX3-F	5′ TTCTTTTCCTCCTCTCTCCTCATC 3′
ZFHX3-R	5′ CGGTCCGTCGGACTTTTG 3′

#### RT-PCR data analysis

The comparative method (ΔΔCt) was used to calculate relative quantities of gene expression levels. To measure the RA-dependent gene expression changes, we used the 24 h pre-treated cells before RA-treatment as the calibrator samples. To estimate the DZNep-induced gene expression changes, we used non-pre-treated cells at time zero as the calibrator samples. For both cases, we used GAPDH as an endogenous control when calculate the ΔCt. Two-tailed t-test was used to estimate the significance.

#### Cell morphology experiment

The LAN5 cells were plated at 40% confluence and allowed to grow for 24 h. DZNep (5uM), vehicle control (DMSO), retinoic acid (RA, 10μM) and DZNep + RA were added and cells were maintained for another 24 h. Cell morphology was examined and pictures were taken using the 20-fold magnification of a Leica DM IRB light microscope.

## Additional files

Additional file 1: Supplementary Tables S1-S4. The analyzed transcriptomic datasets. **Table S2.** The 197 genes harboring verified somatic mutations in high-risk neuroblastoma. Three official gene symbols (WDR85, MLL5, and C12orf69) are updated. **Table S3.** Commonly enriched (FDR < 0.01, intersect > 3) KEGG pathways between the MN_hi genes and the genes harboring verified genetic variants. **Table S4.** DNA-binding extracted from the gene-sets defined by the MSigDB database. (PDF 154 kb)

Additional file 2: Supplementary Figure S1. **Figure S1.** RA-induced cell-dedifferentiation markers in HR-NB over-represent the targets of MYC but not somatic mutations. (PDF 333 kb)

## Abbreviations

DMSO: Dimethyl sulfoxide
DZNep: Depsipeptide, 3-deazaneplanocin A
ESC: Embryonic stem cell
FDR: False discovery rate
FET: Fisher’s exact test
GSP: Gene set-pair
HR-NB: High-risk neuroblastoma
MA: MYCN amplification
MA_hi: up-regulated in MA patients
MN: MYCN normal copy
MN_hi: up-regulated in MN patients
NB: Neuroblastoma
PPI: Protein-protein-interaction
PRC: Polycomb repressive complex
RA: Retinoic acid
RXA-GSP: Relative expression analysis of gene-set pairs
