# Supplementary material for: Incorporating genomic, transcriptomic and clinical data: a prognostic and stem cell-like MYC and PRC imbalance in high-risk neuroblastoma
Source: BMC Syst Biol. 2017 Oct 3;11(Suppl 5):92. doi: 10.1186/s12918-017-0466-5 (PMC5629556; doi:10.1186/s12918-017-0466-5)
Supplement: Supplementary file 1 — Supplementary Tables S1-S4. The analyzed transcriptomic datasets. Table S2. The 197 genes harboring verified somatic mutations in high-risk neuroblastoma. Three official gene symbols (WDR85, MLL5, and C12orf69) are updated. Table S3. Commonly enriched (FDR < 0.01, intersect > 3) KEGG pathways between the MN_hi genes and the genes harboring verified genetic variants. Table S4. DNA-binding extracted from the gene-sets defined by the MSigDB database. (PDF 154 kb) [file 12918_2017_466_MOESM1_ESM.pdf]

Table S1. The analyzed ranscriptomic datasets.

Dataset	Pts size	MA (%)	Pts w. o	OS	EFS	Age	HR w. o	PMID	Used in the manuscript
E-MTAB-161 (AMEXP-1746)	233	36 (15.4)	233	45.1±39	39.4±40	21.4±29	73	20567016	Agilent_meta data to identify MA-signature and train GSP
E-MTAB-179	250	29 (11.6)	250	65.8±33	57.2±37	22.6±32	63	20676065	
GSE27608	47	9 (19.1)	47	40.8±23	NA	34.3±34	27	21501490	exon_meta data to identify MA-signature and train GSP
GSE21713	40	12 (30)	0	NA	NA	NA	0	21145484	
GSE3960	101	20 (19.8)	99	28.3±18	26.4±19	28.6±32	54	21124317	train GSP
GSE16476	88	16 (18.2)	88	83.2±31	75.0±66	17.5±25	39	22367537	train GSP
E-MTAB-161 (AMEXP-1747)	271	35 (12.9)	271	63.9±33	45.7±37	23.8±35	74	20567016	validate GSP

Pts: patient; o: outcome; GSP: gene-set pair; OS: overall survival; EFS: event-free survival

Table S2. The 197 genes harboring verified somatic mutations in high-risk neuroblastoma. Three official gene symbols (WDR85, MLL5,C12orf69) are updated.

Symbol	Entrez Gene Name	Entrez ID	multiple variants	Exp	GSP1	GSP2
ABL2	c-abl oncogene 2, non-receptor tyrosine kinase	27				
AGXT2	alanine--glyoxylate aminotransferase 2	64902	TRUE			
AHCTF1	AT hook containing transcription factor 1	25909	TRUE	MA_hi	Y	
ALK	anaplastic lymphoma receptor tyrosine kinase	238	TRUE	MA_hi	Y	
APOB	apolipoprotein B	338	TRUE			
ARID1A	AT rich interactive domain 1A (SWI-like)	8289	TRUE			Y
ARPP21	cAMP-regulated phosphoprotein, 21kDa	10777				
ASPM	asp (abnormal spindle) homolog, microcephaly associated (Drosophila)	259266				
ATM	ataxia telangiectasia mutated	472	TRUE		Y	
ATRX	alpha thalassemia/mental retardation syndrome X-linked	546	TRUE			
B4GALNT3	beta-1,4-N-acetyl-galactosaminyl transferase 3	283358		MA_hi		
BARD1	BRCA1 associated RING domain 1	580	TRUE	MA_hi	Y	
BEST4	bestrophin 4	266675				
BTN3A2	butyrophilin, subfamily 3, member A2	11118		MN_hi		
C6orf25	chromosome 6 open reading frame 25	80739				
C9orf96	chromosome 9 open reading frame 96	169436				
CACNA1C	calcium channel, voltage-dependent, L type, alpha 1C subunit	775				
CACNA1F	calcium channel, voltage-dependent, L type, alpha 1F subunit	778	TRUE			
CACNA1H	calcium channel, voltage-dependent, T type, alpha 1H subunit	8912		MA_hi		
CACNA1S	calcium channel, voltage-dependent, L type, alpha 1S subunit	779	TRUE			
CACNB3	calcium channel, voltage-dependent, beta 3 subunit	784	TRUE	MN_hi		Y
CALN1	calneuron 1	83698		MA_hi		
CARD11	caspase recruitment domain family, member 11	84433				
CBL	Cbl proto-oncogene, E3 ubiquitin protein ligase	867				
CD247	CD247 molecule	919		MN_hi		
CEACAM5	carcinoembryonic antigen-related cell adhesion molecule 5	1048				
CHD8	chromodomain helicase DNA binding protein 8	57680				
CHEK2	checkpoint kinase 2	11200		MA_hi		
CLCN3	chloride channel, voltage-sensitive 3	1182				
CLTC	clathrin, heavy chain (Hc)	1213		MN_hi		
COL1A1	collagen, type I, alpha 1	1277	TRUE			
COL3A1	collagen, type III, alpha 1	1281	TRUE			
COL4A1	collagen, type IV, alpha 1	1282	TRUE			
COL4A3	collagen, type IV, alpha 3 (Goodpasture antigen)	1285	TRUE			
COL4A5	collagen, type IV, alpha 5	1287				
COL5A1	collagen, type V, alpha 1	1289				
COL5A2	collagen, type V, alpha 2	1290				
COL6A3	collagen, type VI, alpha 3	1293				
COL6A6	collagen, type VI, alpha 6	131873	TRUE			
CREB5	cAMP responsive element binding protein 5	9586	TRUE			
CREBBP	CREB binding protein	1387				
CRX	cone-rod homeobox	1406				
CSMD2	CUB and Sushi multiple domains 2	114784	TRUE			
CSPG4	chondroitin sulfate proteoglycan 4	1464	TRUE			
CTDSPL2	CTD (carboxy-terminal domain, RNA polymerase II, polypeptide A) sma	51496				
CYP11A1	cytochrome P450, family 11, subfamily A, polypeptide 1	1583				
CYP1A1	cytochrome P450, family 1, subfamily A, polypeptide 1	1543	TRUE			

CYP2E1	cytochrome P450, family 2, subfamily E, polypeptide 1	1571				
DDB1	damage-specific DNA binding protein 1, 127kDa	1642				
DEFB115	defensin, beta 115	245929				
DHX58	DEXH (Asp-Glu-X-His) box polypeptide 58	79132		MN_hi		
DKK2	dickkopf WNT signaling pathway inhibitor 2	27123	TRUE			
DNAH5	dynein, axonemal, heavy chain 5	1767	TRUE			
DNAH8	dynein, axonemal, heavy chain 8	1769				
DNM1L	dynamin 1-like	10059				
DOCK1	dedicator of cytokinesis 1	1793				
DPH7	diphthamide biosynthesis 7	92715		MA_hi		
DSC3	desmocollin 3	1825				
DSG4	desmoglein 4	147409	TRUE			
EGF	epidermal growth factor	1950				
EGFR	epidermal growth factor receptor	1956				
EGR1	early growth response 1	1958				
EGR2	early growth response 2	1959				
ELTD1	EGF, latrophilin and seven transmembrane domain containing 1	64123	TRUE			
EP300	E1A binding protein p300	2033				
FANCD2	Fanconi anemia, complementation group D2	2177		MA_hi		
FANCE	Fanconi anemia, complementation group E	2178				
FANCG	Fanconi anemia, complementation group G	2189				
FANCI	Fanconi anemia, complementation group I	55215		MA_hi		
FANCM	Fanconi anemia, complementation group M	57697	TRUE	MA_hi	Y	
FBLN2	fibulin 2	2199				
FCRL6	Fc receptor-like 6	343413	TRUE			
FGFR1	fibroblast growth factor receptor 1	2260				
FLG2	filaggrin family member 2	388698	TRUE			
FREM2	FRAS1 related extracellular matrix protein 2	341640	TRUE			
GABRA6	gamma-aminobutyric acid (GABA) A receptor, alpha 6	2559	TRUE			
GABRQ	gamma-aminobutyric acid (GABA) A receptor, theta	55879	TRUE			
GAL3ST3	galactose-3-O-sulfotransferase 3	89792	TRUE			
GLI3	GLI family zinc finger 3	2737	TRUE			
GLRA3	glycine receptor, alpha 3	8001				
GOT1L1	glutamic-oxaloacetic transaminase 1-like 1	137362				
GPR116	G protein-coupled receptor 116	221395	TRUE			
GRIPAP1	GRIP1 associated protein 1	56850				
GRM6	glutamate receptor, metabotropic 6	2916		MA_hi		
GTPBP1	GTP binding protein 1	9567				
HDDC2	HD domain containing 2	51020		MA_hi		
HIST1H2BD	histone cluster 1, H2bd	3017				
HMCN1	hemicentin 1	83872	TRUE			
HMGCLL1	3-hydroxymethyl-3-methylglutaryl-CoA lyase-like 1	54511				
IGSF11	immunoglobulin superfamily, member 11	152404	TRUE			
IGSF22	immunoglobulin superfamily, member 22	283284	TRUE			
IL16	interleukin 16	3603	TRUE	MN_hi		Y
INPP5D	inositol polyphosphate-5-phosphatase, 145kDa	3635	TRUE	MN_hi		Y
KEAP1	kelch-like ECH-associated protein 1	9817		MA_hi		
KIAA1377	KIAA1377	57562		MN_hi		
KIR3DL1	killer cell immunoglobulin-like receptor, three domains, long cytoplasmic	3811	TRUE			
KMT2E	lysine (K)-specific methyltransferase 2E	55904	TRUE	MN_hi		Y
KNDC1	kinase non-catalytic C-lobe domain (KIND) containing 1	85442		MN_hi		

KRT71	keratin 71	112802	TRUE			
KTN1	kinectin 1 (kinesin receptor)	3895		MN_hi		
LAMA3	laminin, alpha 3	3909	TRUE			
LAMB2	laminin, beta 2 (laminin S)	3913	TRUE			
LILRA3	leukocyte immunoglobulin-like receptor, subfamily A (without TM doma	11026				
LILRB1	leukocyte immunoglobulin-like receptor, subfamily B (with TM and ITIM	10859	TRUE			
LMO1	LIM domain only 1 (rhombotin 1)	4004	TRUE	MN_hi		Y
LRP1B	low density lipoprotein receptor-related protein 1B	53353	TRUE			
LRRTM4	leucine rich repeat transmembrane neuronal 4	80059	TRUE	MN_hi		Y
MAP2K7	mitogen-activated protein kinase kinase 7	5609				
MAPK3	mitogen-activated protein kinase 3	5595		MN_hi		
MARCH6	membrane-associated ring finger (C3HC4) 6, E3 ubiquitin protein ligase	10299		MN_hi		
MBNL3	muscleblind-like splicing regulator 3	55796		MA_hi		
MET	met proto-oncogene	4233	TRUE			
MGA	MGA, MAX dimerization protein	23269	TRUE			
MLH1	mutL homolog 1	4292				
MLLT3	myeloid/lymphoid or mixed-lineage leukemia (trithorax homolog, Drosop	4300				
MOCS1	molybdenum cofactor synthesis 1	4337				
MOSPD3	motile sperm domain containing 3	64598				
MRPS27	mitochondrial ribosomal protein S27	23107	TRUE	MA_hi	Y	
MSH2	mutS homolog 2	4436	TRUE	MA_hi	Y	
MSR1	macrophage scavenger receptor 1	4481				
MTTP	microsomal triglyceride transfer protein	4547				
MYCN	v-myc avian myelocytomatosis viral oncogene neuroblastoma derived hor	4613	TRUE	MA_hi	Y	
NCAM1	neural cell adhesion molecule 1	4684	TRUE	MN_hi		Y
NCAN	neurocan	1463	TRUE	MA_hi	Y	
NCBP1	nuclear cap binding protein subunit 1, 80kDa	4686		MA_hi		
NF1	neurofibromin 1	4763	TRUE			
NFKB1	nuclear factor of kappa light polypeptide gene enhancer in B-cells 1	4790				
NFKB2	nuclear factor of kappa light polypeptide gene enhancer in B-cells 2 (p49/	4791				
NMBR	neuromedin B receptor	4829				
NOTCH1	notch 1	4851	TRUE			
NRAS	neuroblastoma RAS viral (v-ras) oncogene homolog	4893	TRUE	MN_hi		Y
OR14A16	olfactory receptor, family 14, subfamily A, member 16	284532	TRUE			
OR5L1	olfactory receptor, family 5, subfamily L, member 1	219437	TRUE			
PALB2	partner and localizer of BRCA2	79728		MA_hi		
PANX1	pannexin 1	24145	TRUE			
PBRM1	polybromo 1	55193	TRUE			
PDCD1LG2	programmed cell death 1 ligand 2	80380				
PDGFA	platelet-derived growth factor alpha polypeptide	5154				
PDGFRA	platelet-derived growth factor receptor, alpha polypeptide	5156		MA_hi		
PGLYRP3	peptidoglycan recognition protein 3	114771	TRUE			
PIK3C2G	phosphatidylinositol-4-phosphate 3-kinase, catalytic subunit type 2 gamm	5288				
PIK3CA	phosphatidylinositol-4,5-bisphosphate 3-kinase, catalytic subunit alpha	5290	TRUE			
PIK3CB	phosphatidylinositol-4,5-bisphosphate 3-kinase, catalytic subunit beta	5291	TRUE			
PKHD1	polycystic kidney and hepatic disease 1 (autosomal recessive)	5314	TRUE			
PLAG1	pleiomorphic adenoma gene 1	5324	TRUE			
PLCB1	phospholipase C, beta 1 (phosphoinositide-specific)	23236				
PLCG1	phospholipase C, gamma 1	5335		MA_hi		
PLK4	polo-like kinase 4	10733		MA_hi		
PLXNB2	plexin B2	23654	TRUE			

PMFBP1	polyamine modulated factor 1 binding protein 1	83449	TRUE			
PPP2CB	protein phosphatase 2, catalytic subunit, beta isozyme	5516				
PRIM2	primase, DNA, polypeptide 2 (58kDa)	5558	TRUE			
PRKACG	protein kinase, cAMP-dependent, catalytic, gamma	5568	TRUE			
PRKCQ	protein kinase C, theta	5588				
PTK2	protein tyrosine kinase 2	5747				
PTK6	protein tyrosine kinase 6	5753				
PTPN11	protein tyrosine phosphatase, non-receptor type 11	5781	TRUE			
PTPN6	protein tyrosine phosphatase, non-receptor type 6	5777		MN_hi		
PYHIN1	pyrin and HIN domain family, member 1	149628	TRUE			
RAD54B	RAD54 homolog B (S. cerevisiae)	25788				
RAD54L2	RAD54-like 2 (S. cerevisiae)	23132				
RAF1	v-raf-1 murine leukemia viral oncogene homolog 1	5894		MA_hi		
RMI1	RecQ mediated genome instability 1	80010				
RPL7L1	ribosomal protein L7-like 1	285855				
RPS6KA3	ribosomal protein S6 kinase, 90kDa, polypeptide 3	6197				
S100A14	S100 calcium binding protein A14	57402	TRUE			
SGSM1	small G protein signaling modulator 1	129049	TRUE			
SH3GL2	SH3-domain GRB2-like 2	6456				
SHC1	SHC (Src homology 2 domain containing) transforming protein 1	6464		MN_hi		
SLC6A7	solute carrier family 6 (neurotransmitter transporter), member 7	6534	TRUE			
SMAD4	SMAD family member 4	4089				
SMCO3	single-pass membrane protein with coiled-coil domains 3	440087				
SPHKAP	SPHK1 interactor, AKAP domain containing	80309	TRUE			
SPRR1A	small proline-rich protein 1A	6698	TRUE			
SPTA1	spectrin, alpha, erythrocytic 1 (elliptocytosis 2)	6708	TRUE			
SPTBN1	spectrin, beta, non-erythrocytic 1	6711		MN_hi		
SPTBN4	spectrin, beta, non-erythrocytic 4	57731		MN_hi		
SRA1	steroid receptor RNA activator 1	10011				
STAB2	stabilin 2	55576	TRUE			
STAG1	stromal antigen 1	10274	TRUE	MA_hi	Y	
STAM2	signal transducing adaptor molecule (SH3 domain and ITAM motif) 2	10254		MN_hi		
SUCLG2	succinate-CoA ligase, GDP-forming, beta subunit	8801				
SYNRG	synergin, gamma	11276	TRUE	MN_hi		Y
TJP3	tight junction protein 3	27134	TRUE			
TLN2	talin 2	83660	TRUE	MN_hi		Y
TP53	tumor protein p53	7157		MA_hi		
TSGA10IP	testis specific, 10 interacting protein	254187	TRUE			
TTC24	tetratricopeptide repeat domain 24	164118				
TTN	titin	7273	TRUE			
USP48	ubiquitin specific peptidase 48	84196		MN_hi		
WDR11	WD repeat domain 11	55717		MN_hi		
WDR73	WD repeat domain 73	84942				
ZC3HC1	zinc finger, C3HC-type containing 1	51530	TRUE			
ZNF44	zinc finger protein 44	51710				
ZNF529	zinc finger protein 529	57711	TRUE			
ZNF57	zinc finger protein 57	126295				
ZNF780B	zinc finger protein 780B	163131	TRUE			

Table S3. Commonly enriched (FDR<0.01, intersect>3) KEGG pathways beteen MN_hi genes and genes harboring verified genetic variants.

KEGG GeneSet	Odds.MN	FDR.MN	Intersect.MN	Odds.muy	FDR.mut	Intersect.mut	Intersect_gene.common
PROSTATE CANCER	2.3	5.9E-03	23	13.4	4.6E-10	15	NRAS, MAPK3
GLIOMA	3.2	4.5E-04	21	14.4	1.3E-08	12	SHC1, NRAS, MAPK3
NEUROTROPHIN SIGNALING PATHWAY	3.3	1.1E-06	41	6.6	9.1E-06	12	NRAS, SHC1, MAPK3
MAPK SIGNALING PATHWAY	2.0	1.2E-04	61	5.6	1.8E-07	20	NRAS, CACNB3, MAPK3
ERBB SIGNALING PATHWAY	2.7	9.1E-04	25	11.3	2.6E-08	13	SHC1, NRAS, MAPK3
ENDOMETRIAL CANCER	4.2	6.6E-05	20	12.9	1.5E-06	9	NRAS, MAPK3
T CELL RECEPTOR SIGNALING PATHWAY	2.5	7.8E-04	29	8.9	2.6E-07	13	NRAS, CD247, PTPN6, MAPK3
ADHERENS JUNCTION	3.9	9.3E-06	27	7.4	1.1E-04	8	PTPN6, MAPK3
GAP JUNCTION	3.3	1.6E-05	30	6.8	7.8E-05	9	NRAS, MAPK3
B CELL RECEPTOR SIGNALING PATHWAY	3.2	1.8E-04	24	8.5	2.0E-05	9	PTPN6, INPP5D, NRAS, MAPK3
GNRH SIGNALING PATHWAY	2.7	3.3E-04	29	6.8	3.6E-05	10	NRAS, MAPK3
NON SMALL CELL LUNG CANCER	2.8	7.0E-03	16	12.3	1.9E-06	9	NRAS, MAPK3
LONG TERM POTENTIATION	2.9	1.1E-03	21	9.2	1.3E-05	9	NRAS, MAPK3
COLORECTAL CANCER	3.2	5.9E-04	20	9.0	4.0E-05	8	MAPK3
FC GAMMA R MEDIATED PHAGOCYTOSIS	3.4	9.3E-06	32	4.7	2.9E-03	7	INPP5D, MAPK3
PANCREATIC CANCER	2.6	2.9E-03	20	9.0	1.3E-05	9	MAPK3
RENAL CELL CARCINOMA	2.5	8.0E-03	19	9.0	1.3E-05	9	NRAS, MAPK3
NATURAL KILLER CELL MEDIATED CYTOTOXICITY	2.2	2.9E-03	32	5.7	5.2E-05	11	NRAS, CD247, SHC1, PTPN6, MAPK3
CHEMOKINE SIGNALING PATHWAY	2.4	4.8E-05	49	3.4	3.3E-03	10	NRAS, SHC1, MAPK3
FC EPSILON RI SIGNALING PATHWAY	2.7	9.6E-04	23	6.8	1.9E-04	8	INPP5D, NRAS, MAPK3
REGULATION OF ACTIN CYTOSKELETON	2.1	2.9E-04	50	3.7	7.8E-04	12	NRAS, MAPK3
INSULIN SIGNALING PATHWAY	2.4	3.2E-04	36	4.3	1.4E-03	9	NRAS, SHC1, INPP5D, MAPK3
ACUTE MYELOID LEUKEMIA	3.3	5.6E-04	19	7.0	1.1E-03	6	NRAS, MAPK3
PHOSPHATIDYLINOSITOL SIGNALING SYSTEM	3.1	3.6E-04	24	5.1	3.9E-03	6	INPP5D
BLADDER CANCER	2.9	9.1E-03	13	9.9	2.3E-04	6	NRAS, MAPK3
APOPTOSIS	2.4	4.7E-03	23	4.4	6.6E-03	6	

Table S4. DNA-binding extracted from the gene-sets defined in the MSigdb dataset.

MsigDB _ID	description; motif at the promoter regions [-2kb,2kb] around transcription start site	Pubmed	# of genes
**MYCN targets**			
LASTOWSKA_COAMPLIFIED_WITH_MYCN	Genes co-amplified within MYCN in primary neuroblastoma tumors	17533364	43
KIM_MYCN_AMPLIFICATION_TARGETS_UP	Genes positively correlated with amplifications of MYCN in small cell lung cancer cell lines.	16116477	92
KIM_MYCN_AMPLIFICATION_TARGETS_DN	Genes negatively correlated with amplifications of MYCN in small cell lung cancer cell lines.	16116477	103
WEI_MYCN_TARGETS_WITH_E_BOX	Genes whose promoters contain E-box motifs and whose expression changed in MYCN-3 cells (neuroblastoma) upon induction of MYCN	18504438	795
V$NMYC_01	NNCCACGTGNNN for MYCN		271
Sum of unique genes			1259
**PRC2 targets in ES cells**			
BENPORATH_PRC2_TARGETS	PRC2 targets identified by ChIP on chip on human ESC as genes that posess the trimethylated H3K27 mark in their promoters and are bound by SUZ12 and EED	18443585	652
BENPORATH_EED_TARGETS	Genes identified by ChIP on chip as targets of the Polycomb protein EED in human ESC	18443585	1062
BENPORATH_SUZ12_TARGETS	Genes identified by ChIP on chip as targets of the Polycomb protein SUZ12 in human ESC	18443585	1038
BENPORATH_ES_WITH_H3K27ME3	Genes posessing the H3K27me3 mark in their promoters in human ESC as identified by ChIP on chip.	18443585	1118
Sum of unique genes			1587
**EZH2 inhibited targets in solid cancer cells**			
NUYTTEN_EZH2_TARGETS_UP	Genes up-regulated in PC3 cells (prostate cancer) after knockdown of EZH2 by RNAi.	17724462	1037
KONDO_EZH2_TARGETS	Genes up-regulated in PC3 cells (prostate cancer) after EZH2 knockdown by RNAi.	18488029	245
LU_EZH2_TARGETS_UP	Genes up-regulated in SKOV3ip1 cells (ovarian cancer) upon knockdown of EZH2 by RNAi.	20708159	295
Sum of unique genes			1520
**MYC/MYCN targets with motif match**			
V$MYCMAX_01	NNACCACGTGGTNN for MYC		255
V$MYCMAX_02	NANCACGTGNNW for MYC		268
V$MYCMAX_B	GCCAYGYGSN for MYC		268
V$MYCMAX_03	NNNNNNNCACGTGNNNNNNN for MYC		252
V$MYC_Q2	CACGTGS for MYC		185
CACGTG_V$MYC_Q2	CACGTG for MYC		1032
V$NMYC_01	NNCCACGTGNNN for MYCN		271
Sum of unique genes			1777
