# Supplementary material for: Incorporating genomic, transcriptomic and clinical data: a prognostic and stem cell-like MYC and PRC imbalance in high-risk neuroblastoma
Source: BMC Syst Biol. 2017 Oct 3;11(Suppl 5):92. doi: 10.1186/s12918-017-0466-5 (PMC5629556; doi:10.1186/s12918-017-0466-5)
Supplement: Supplementary file 2 — Supplementary Figure S1. Figure S1. RA-induced cell-dedifferentiation markers in HR-NB over-represent the targets of MYC but not somatic mutations. (PDF 333 kb) [file 12918_2017_466_MOESM2_ESM.pdf]

Supplementary Figures

Incorporating genomic, transcriptomic and clinical data: A prognostic and stem cell-like MYC and PRC imbalance in high-risk neuroblastoma

Xinan Holly Yang*, Fangming Tang, Jisu Shin, John M Cunningham*

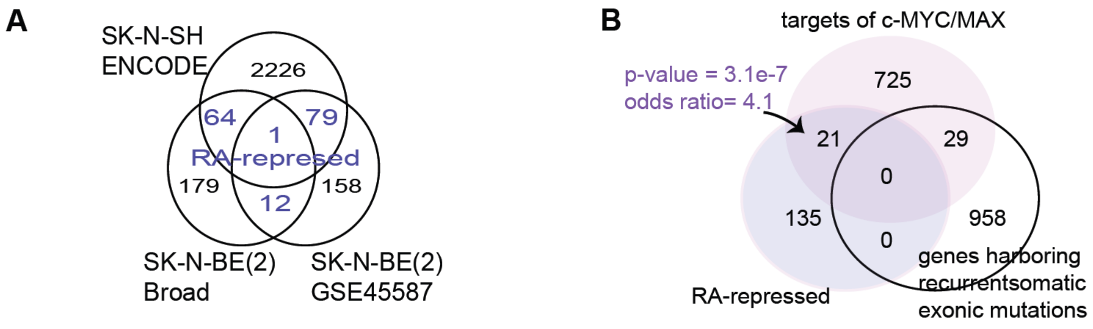

Figure S1. RA-induced cell-dedifferentiation markers in HR-NB over-represent the targets of MYC but not somatic mutations. **a** Venn diagrams demonstrating how to identify the core set of RA-repressed dedifferentiation markers. **b** Venn diagram of the gene targets of MYC/MAX, the RA-repressed genes, and the genes harboring recurrent somatic mutations at exon in HR-NB. Significance of enrichment is estimated using the Fisher's exact test against approximately 21,000 human genes.
